# Phage display library selection of a hypoxia-binding scFv antibody for liver cancer metabolic marker discovery

**DOI:** 10.18632/oncotarget.9460

**Published:** 2016-05-18

**Authors:** Jing Liu, Qing Zhang, Hang Chen, Zhihui Gao, Yao Li, Zhongyuan Sun, Rong Xiang, Sihe Zhang

**Affiliations:** ^1^ Department of Medical Biochemistry and Cell Biology, School of Medicine, Nankai University, Tianjin, China; ^2^ Department of Clinical Laboratory, Cancer Hospital of Tianjin Medical University, Tianjin, China; ^3^ MOE Key Laboratory of Bioinformatics, School of Life Sciences, Tsinghua University, Beijing, China

**Keywords:** hypoxia, phage antibody library, M2 splice isoform of pyruvate kinase, biomarker, liver cancer

## Abstract

Hypoxia, which is frequently observed in liver cancer and metastasis, influences tumor progression and resistance to therapy. Although hypoxia-associated biomarkers are of use in other cancers, none is recognized as a surrogate for hypoxia in liver cancer. In this study, we generated seven unique human single-chain Fv (scFv) antibodies (Abs) specific to hypoxic liver cancer cells, using normoxia-depleted vs hypoxia-selected phage library panning technology. By developing the scFv immunoprecipitation-based mass spectrometry method, the antigen that bound with one of the Abs (H103) was identified as the M2 splice isoform of pyruvate kinase (PKM2), an enzyme that is a key regulator of aerobic glycolysis in cancer cells. Increased expression of PKM2 was induced by hypoxia in liver cancer cell lines. Immunohistochemical (IHC) staining showed that PKM2 was highly expressed in moderately and well differentiated hepatocellular carcinoma (HCC) tissues with a hypovascular staining pattern. High expression of PKM2 was also localized in the perinecrotic area of intrahepatic cholangiocarcinoma (ICC) tissues. The percentage of the HCC or ICC tumor expressing PKM2 was significantly higher with more tumor necrosis, low microvessel density, and advanced stage. Moreover, the H103 scFv Ab was efficiently internalized into hypoxic liver cancer cells and could have potential for targeted drug delivery. Conclusion: our study, for the first time, developed hypoxia-specific scFv Ab H103 to liver cancer cells, and revealed that PKM2 is a promising biomarker for hypoxia in HCC and ICC tissues. These allow further exploration of this valuable Ab and PKM2 antigen for hypoxia targeting in liver cancer.

## INTRODUCTION

Hypoxia is not only considered to be a hallmark of cancer which promotes angiogenesis, invasiveness, and metastasis, but also has a major role in metabolic reprogramming of cancer cells which allows cells to survive in a hostile secondary metastatic environment [1]. Moreover, tumor self-renewal, metastatic potential, and chemo- and radiotherapy resistance are thought to be driven by a population of cancer stem cells (CSC), which remain dormant in hypoxic microenvironmental niches [2]. Because of its central role in tumor progression and resistance to therapy, cancer cell adaptation to hypoxia has been used to develop tumor markers and target therapies [3].

Although the liver is a hypervascular organ, severe hypoxia is always found inside hepatocellular carcinoma (HCC) due to its rapid growth nature. HCC is generally developed through cirrhosis, which always causes fibrogenesis which demolishes the normal blood system, leads to a shortage of blood circulation and finally results in hypoxia. Moreover, conventional palliative therapies for HCC including hepatic artery ligation (HAL), trans-catheter arterial embolization (TAE), and trans-catheter arterial chemoembolization (TACE) can also cause hypoxia. Although the principle of these palliative therapies is to restrict blood supply to suppress tumor growth, these therapies which induce hepatic ischemia always catastrophically result in hypoxia. No biomarkers, such as the hypoxia inducible factor (HIF), carbonic anhydrase (CA), glucose transporter (GLUT), monocarboxylate transporters (MCT), and osteopontin, which are recognized as surrogates for hypoxia in other cancers [3–7], can reflect the hypoxic status in HCC. Although scattered studies demonstrated ‘successful’ detection of HIF-1α and HIF-2α in HCC specimens [8, 9], because of the extremely short half-life of these proteins, it is hard to precisely assess the level of hypoxia in practical clinic situations. Thus, discovery of novel hypoxia-specific markers for HCC targeted therapies is urgently needed.

Phage display technology can be used to isolate a specific antibody (Ab) able to bind its cognate antigen or to internalize into tumor cells [10–15]. For the Ab phage display, Ab fragments, corresponding to the binding site of an immunoglobulin (Ig) either in a single-chain Fv (scFv) or in an antigen-binding fragment (Fab) format are fused to the pIII minor capsid protein and displayed at the surface of the M13 filamentous phage [16–18]. Repertoires of human Ab variable (V) genes can be cloned and used to construct a large Ab library, which can then be used to select large panels of Abs to virtually any antigen. Direct selection from the phage Ab library on tumor cells provides a straightforward approach for generating human Abs that recognizes tumor specific markers [12, 14, 19–21]. However, this approach often yields Abs binding to a wide range of undesired common cell surface proteins. Optimized methods can be employed to focus the specificity of the resultant clones on certain molecular or cellular phenotypes. These include performing a subtraction selection against normal cells to deplete undesired clones prior to panning against the tumor cells of interest, or stripping unwanted phages from the cell surface prior to isolating those that bind the internalized antigens [10–14].

In this study, we employed the panning approach combining non-tumor cell depletion with normoxia subtraction, to select against a human scFv Ab phage library. This approach efficiently generated a panel of phage scFv Abs that specifically bind to liver cancer cells only under hypoxic conditions. By developing a scFv-based immunoprecipitation method, we were able to use liquid chromatography tandem mass spectrometry (LC–MS/MS) to identify the antigen bound by one of the Abs (H103) as the M2 splice isoform of pyruvate kinase (PKM2), an enzyme that is a key regulator of aerobic glycolysis (known as the Warburg effect) in cancer cells [22–24]. Increased expression of the PKM2 protein is induced by hypoxia in liver cancer cell lines, especially in high metastatic cell lines. Immunohistochemical (IHC) staining with the H103 scFv Ab on tissue arrays indicated that PKM2 is highly expressed in moderately and well differentiated HCC tissues with a hypovascular staining pattern. High expression of PKM2 is also localized in the perinecrotic area of intrahepatic cholangiocarcinoma (ICC) tissue. The percentage of PKM2 expression in liver cancers is significantly higher with more tumor necrosis, low microvessel density (MVD), and advanced stage. Moreover, the H103 scFv Ab is specifically internalized into hypoxic HCC cells and could have potential for targeted drug delivery. Our study developed, for the first time, a hypoxia specific scFv Ab binding to HCC by phage library selection, and revealed that its antigen PKM2 is a potential biomarker for hypoxia in liver cancer tissues.

## RESULTS

### Human scFv Ab phage display library

A large human scFv Ab phage library was created from the Ig variable domains of 9 liver cancer patients. Patients received different treatments, including HAL, TAE, and TACE, and were specifically incorporated into the library in an attempt to enhance the diversity of the hypoxia-responding Ig gene. The prime scFv library contains an estimated 1.5 × 10^9^ cfu transformants. DNA sequencing predicted that the library contains 93% full-length scFv inserts with approximately 89% of the total clones encoding functional open reading frames (Figure S1A). Germline gene alignment analysis showed that five out of the seven VH gene families were present. VH3 comprised 39% of the clones with VH4 and VH2 representing an additional 21% of the clones. The remainders of the clones were comprised of VH1 and VH6 (Figure S1B). This diversity is also presented for Vκ and Vλ gene families in our scFv library (Figure S1B).

### Normoxia-depleted vs hypoxia-selected library panning

**Figure 1:**
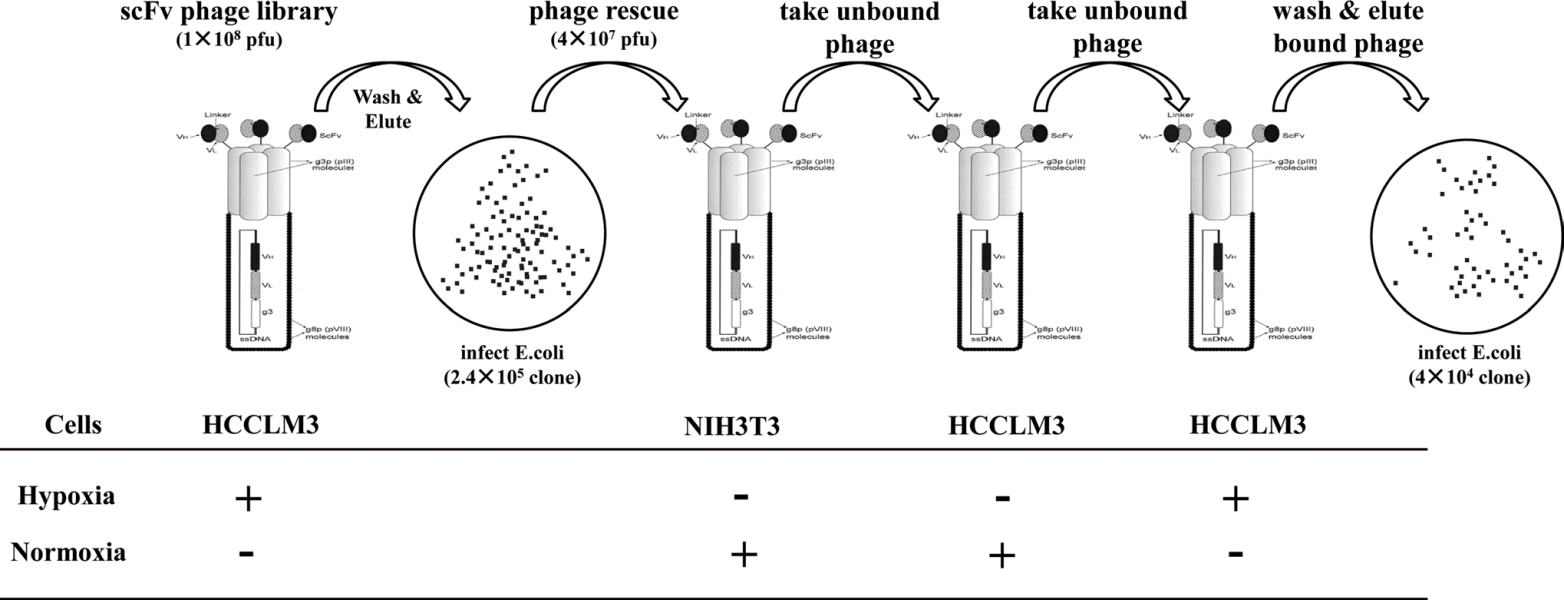
Panning procedure of the human scFv Ab phage display library For each round of panning of the rescued phage library, sequential selections were carried out: positive selections (+) were performed on hypoxic treated HCCLM3 cells at the first and last steps of panning. After washing with PBST and stripping by a glycine/NaCl buffer, the hypoxic bound phages were re-infected for propagation and forwarded to subtractive selections (−) on normoxic cultured 3T3 cells (step two) and HCCLM3 cells (step three). The resultant sub-library was rescued again and subjected to the next round of panning.

**Figure 2:**
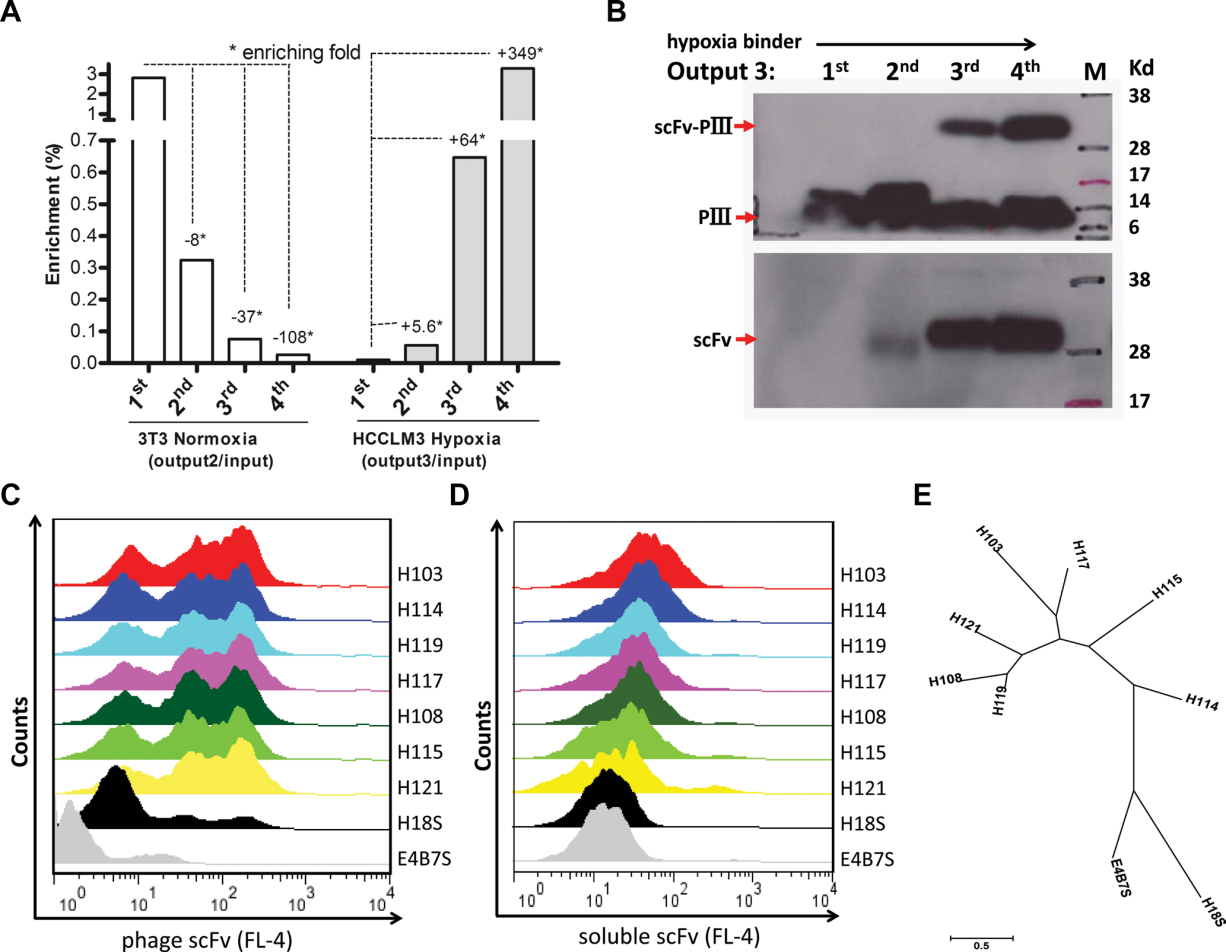
Panning and screening of hypoxia-binding scFv Abs (**A**) Binding of the scFv library to hypoxic (Right) vs normoxic (Left) cells was determined by phage titer assay. Output/Input ratios for every rounds of panning are indicative of enrichment folds on the selected cells. (**B**) The pIII-scFv display level (Up) and soluble scFv expression level (Low) of hypoxic binding sub-libraries from rounds of panning were analyzed by Western blotting with the anti-M13 Ab (up, 1:2000, Amersham) and anti-his tag Ab (low, 1:2000, Sungene Biotech), respectively. Hypoxic binding activity of rescued phage supernatants (**C**) and IPTG-induced supernatant concentrations (**D**) from seven selected clones were analyzed by flow cytometer assay on HCCLM3 cells. H18S scFv, negative control. E4B7S scFv, unrelated control. (**E**) Phylogenetic tree of seven unique hypoxia binding scFv Abs was built up using MEGA 6.06 software. The neighbor-joining statistical method was utilized on the maximum composite likelihood model. The unit of measure (scale bar) represents the number of amino acid substitutions per site.

High background and low yield of positive clones are generally associated with conventional cell-based panning methods. We first validated that our panning was independent of phage binding and optimized the stripping condition to increase the yield of bound phage clones. We found that the rescued phage scFv library more markedly bound than a helper phage to HCCLM3 cells (Figure S2A), and three times washing (0.5 min per time) with 100 mM glycine/0.5 M Nacl was the best stripping condition for selection (Figure S2B). It was reported that cell-based panning typically results in 1–12 phages bound per cell [11]. Based on this criterion, we reasoned that a minimum of 10^7^ HCCLM3 cells would be sufficient to isolate all hypoxia-binders during the first and last steps of selections in each round of panning. For the sake of completely depleting the unspecific binders on the cell surface, we carried out sequential depletions in the second and third steps of each round of panning by incubating a 4 × 10^7^ pfu phage library with a 10-fold excess of nomoxic NIH3T3 and HCCLM3 cells (Figure 1). Despite variations both in the absolute ratio of hypoxic to normoxic cells and in the washing stringency used, 64 and 349 fold enrichments were respectively obtained in the last two rounds of panning (Figure 2A). Significant enrichment was also observed on the levels of the scFv Ab phage display and soluble expression (Figure 2B). These results indicated a selective enrichment of the hypoxia-bound phage clones after four rounds of panning.

### Selection of hypoxia binding scFv Abs

Hypoxia binders to HCCLM3 cells were initially screened by mono-phage based flow cytometric evaluation. 32 out of 90 clones from the last round that displayed binding values that were three- to ten-fold above the background control were selected. These candidate clones in their soluble scFv form were further evaluated by flow cytometer analysis, and 24 clones that markedly bound to hypoxia HCCLM3 cells were validated. DNA sequencing analysis further revealed seven unique full-length scFv clones and the remaining seventeen clones encoding truncated scFvs (data not shown) were removed from further evaluation. There was a predominance of VH germline VH4-4 and VH3-23 usage that recombined with four different JH segments to form the rearranged heavy chain genes. Likewise, the Vl germline V6-57 gene was predominantly used in combination with Jl2-3 segments (Table S1). A phylogenetic tree based on the amino acid sequence of these seven scFv Abs is presented (Figure 2E). The IMGT/V-QUEST alignment showed that the VH and VL genes of the selected H103 scFv Ab (GeneBank: KP347981.1) possess high homology to the human IGHV4-4*03 and IGLV6-57*02 germline genes, respectively (Table 1). Since the H103 scFv Ab presented the strongest hypoxia-binding activity among these positive clones (Figure 2C, 2D), we focused on its identifying characters in the remained of this study.

**Table 1:** IMGT-VQUEST sequence analysis of hypoxia-binding scFv Ab H103

GPAGHGPGAAAESGPGLVRPSGTLSLICAVS**GDSISSSIW**WSWVRQSPGKGLEWIGY**IYHNGNT**YYNPSLESRVTIS VDTSENQFSLKLSSVTAADTAVYYC**ARGYDSSGYYWTDDRYYFDY**WGQGTLVTVSSGGGGSGGGGSGGGGS NFMLTQPHSVSESPGKTVTISCTGS**SGSIASNY**VQWYQQRPGSAPTTVIYEDNQRPPGVPDRFSGSIDSSSNSASL TISALETEDEADYYC**QSYDSRNIDVV**FGGGTKVTVLGAAA**HHHHHH**

Note: Hatching: FR1, FR2, FR3, FR4; Double underline: CDR1, CDR2, CDR3; Undee: (G4S)3 inter-domain linker; Double underline: 6 × his tag.

### Characterization of a hypoxia-specific scFv H103

**Figure 3:**
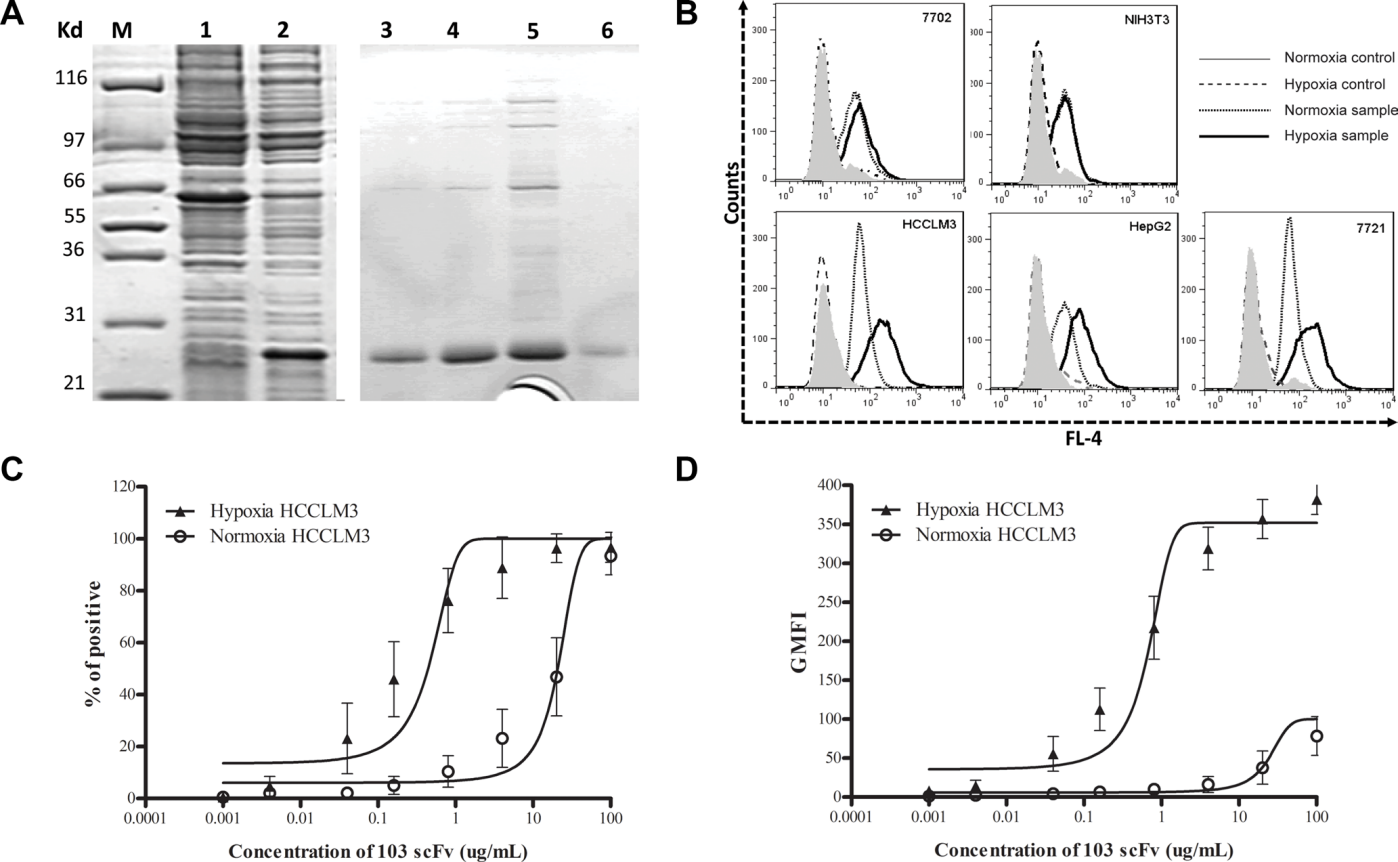
Characterization of the hypoxia-binding H103 scFv Ab (**A**) Coomassie blue staining on SDS-PAGE gels loaded with whole cell lysate of pET28a/103scFv-transfected BL21(DE3) E.coli culture with (lane 1) or without (lane 2) 0.8 mM IPTG for 8 h induced expression, and loaded with the purified H103 scFv Ab after concentration-gradient diluting with imidazole (lane 3–6: 50, 100, 300, or 500 mM). (**B**) The H103 scFv Ab was incubated with liver cancer cells followed by staining with a mixture of mouse anti-his McAb and AF647 conjugated goat anti-mouse pcAb. The binding activity was examined by flow cytometric analysis. Data is presented as overlapping histograms of a hypoxix vs normoxic binding signal, with secondary and tertiary Abs settings as controls. (**C**, **D**) The H103 scFv Ab at the concentrations indicated on the X-axis were respectively incubated with 4.5 × 10^5^ hypoxic or normoxic treated cells followed by staining with secondary anti-His and tertiary AF647 conjugate. The binding activity was analyzed by a flow cytometer and shown as both percentages of positive cells (C) and absolute geometric mean fluorescence intensity (GMFI) (D). The affinity of the scFv Ab binding to hypoxic or normoxic HCCLM3 cells was determined from a non-linear fit of the relative fluorescence intensity to concentration of the bound scFv Ab. Data used included the following: mean ± SEM (error bars), *n* = 3, with ≥ 20,000 cells counted per sample.

A soluble H103 scFv bearing (His) 6 tag was produced and purified using immobilized metal affinity chromatography (Figure 3A). The H103 scFv Ab had much stronger binding to hypoxic HCCLM3, HepG2, and 7721 cells than to their normoxic cultures, while this sharp contrast of bindings was not observed on normal liver cell 7702 and non-tumor NIH3T3 cells (Figure 3B). We also compared the hypoxia binding of the H103 scFv Ab with the hypoxia-specific marker Ab GT12, liver cancer specific scFv Ab H18S, and prostate cancer specific scFv Ab E4B7S. Both H103 scFv and GT12 IgG could markedly recognize the hypoxic liver cancer cells (binding activity: HCCLM3 > HepG2 > 7721), but not their normoxic cultures. In contrast, H18S scFv only weakly bound under hypoxic conditions and E4B7S scFv showed no binding (Table S2). Next, the relative affinity of the H103 scFv Ab was determined by flow cytometric saturation binding assay using serial dilutions of scFv to stain the cells. The EC50 values to reach 50% of maximum percentage positive binding was 0.44 ug/mL for hypoxic HCCLM3 cells but 20.67 ug/mL for normoxic ones. The EC50 values to half-maximal GMFI also showed a sharp-contrast, with 0.64 ug/mL for hypoxic cells and 24.12 ug/mL for normoxic ones (Figure 3C, 3D). The calculated apparent binding constants (Ka) of the H103 scFv Ab to hypoxic cells was 2.08 × 10^8^ M^−1^, which is around three fold higher than that to normoxic cultures. These results indicate that the selected human scFv Ab H103 specifically binds to hypoxic liver cancer cells.

### Evaluation of the internalization property of the H103 scFv Ab

**Figure 4:**
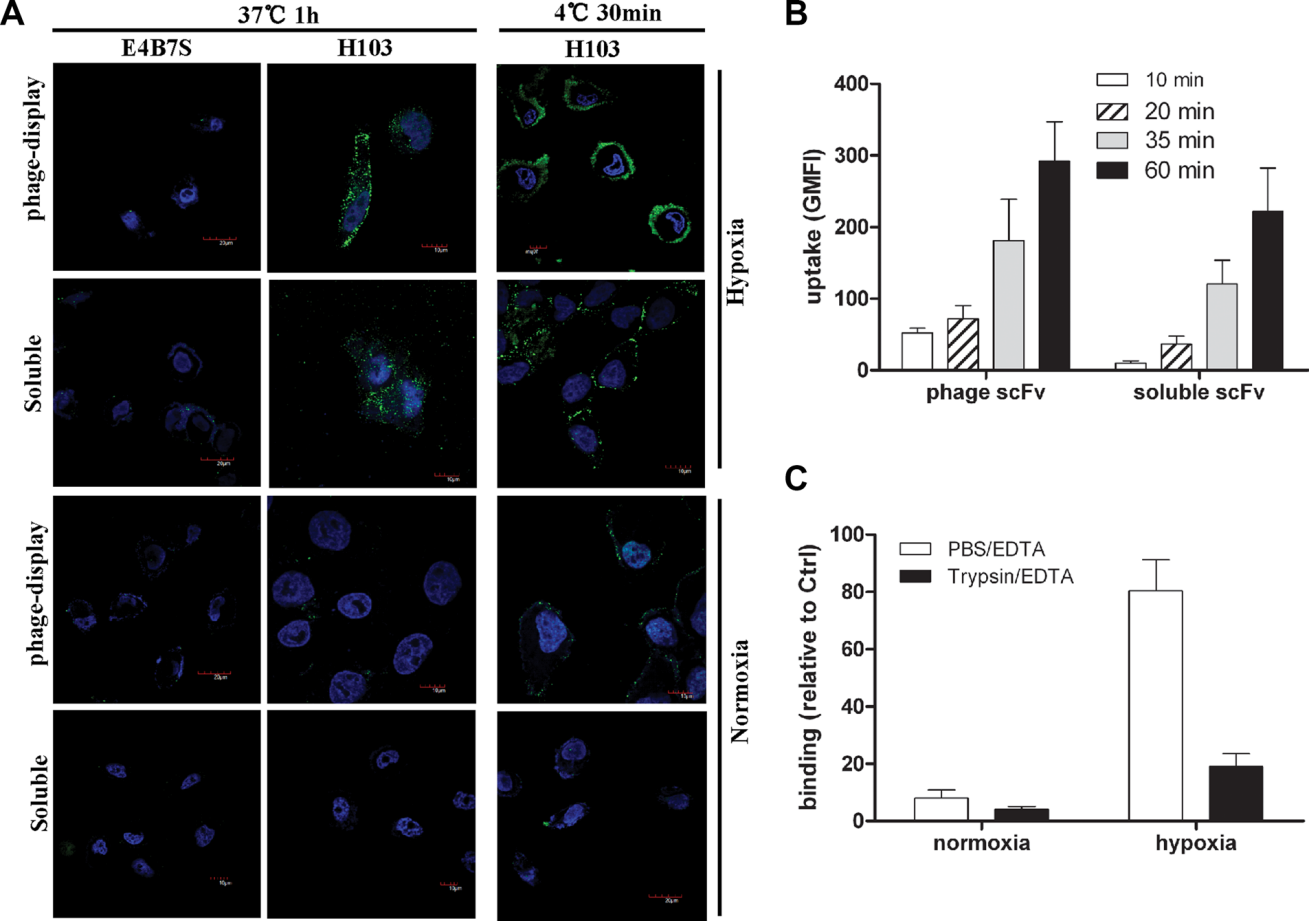
Internalization and binding analysis of the H103 scFv Ab (**A**) Normoxic or hypoxicc treated HCCLM3 cells were incubated with H103 scFv Ab either in phage-display form or soluble form. After washing with PBST, the binding and uptake of the H103 scFv Ab was detected with AF488 conjugated anti-M13 (PVIII) or anti-His Abs under a confocal microscope. E4B7S scFv was used as the control. (**B**) The uptakes of the H103 scFv Ab at different time points in hypoxic HCCLM3 cells were measured by flow cytometric analysis. (**C**) After detachment with PBS/EDTA or T/E, the binding of the H103 scFv Ab on HCCLM3 cells was analyzed by flow cytometry.

Under normoxic conditions, the H103 phage Ab gave no intracellular signal with only tiny heterogeneous cell surface staining. In contrast, both strong cell surface staining and intracellularly homogeneous localization of H103 phage particles are observed in hypoxic cells, demonstrating an efficient uptake under hypoxic conditions (Figure 4A). Similar internalization patterns were observed for the soluble H103 scFv Ab in hypoxic cells, and it presented a stronger intracellular signal with relatively less cell surface residual binding after the uptake (Figure 4A). No uptake signal was observed for E4B7 scFv, and only a minimal intracellular signal was detected for H18s scFv (data not show). We also tested the time-course dynamic uptake of the H103 scFv Ab by flow cytometric measurement. Hypoxia-specific uptakes were detected as soon as 10 minutes after the application of the H103 phage scFv, and 20 minutes after the soluble H103 scFv was applied (Figure 4B). In addition, the hypoxic binding of the H103 scFv Ab was remarkably impaired by Trypsin/EDTA detachment (Figure 4C). These results demonstrated the hypoxia-specific internalization of the H103 scFv Ab in liver cancer cells.

### Identification of the antigen bound with the H103 scFv Ab

**Figure 5:**
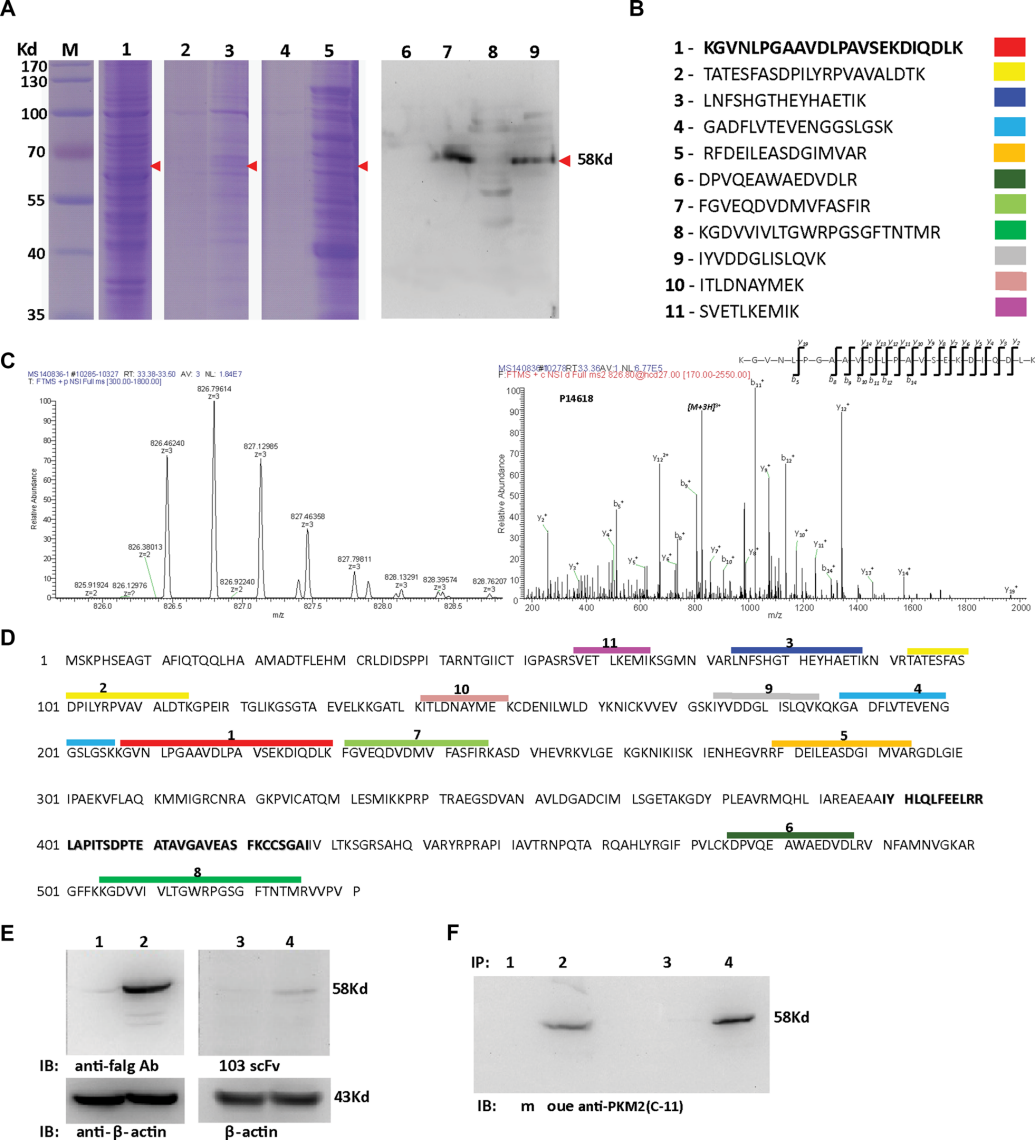
Identification of the antigen bound with the H103 scFv Ab (**A**) H103 scFv (his-tag) coupled protein L (lane 3, 7) or Ni-NTA-agaroses (lane 5, 9) were used to precipitate the hypoxic lysate after HCCLM3 cell surface biotinylation. Total cell lysate (lane 1), immunoprecipitates only with protein L (lane 2, 6), or only with Ni-NTA-agarose (lane 4, 8) were used as controls. Immune complexes, after 4× RIPA buffer washing (lane 2, 3, 6, 7) or eluted using 200 mM imidazole (lane 4, 5, 8, 9), were loaded for SDS-PAGE electrophoresis followed by Coomassie blue staining (lane 1-5) or tested by Western blot using HRP conjugated streptavidin (lane 6–9). (**B**) The same-size bands (between lane 3, 5 and lane 7, 9) from H103 scFv Ab immunoprecipitations were subjected to in gel digestion and 11 characteristic peptides were presented by LC–MS/MS; (**C**) A MS/MS spectrum of a doubly charged TMT-labeled peptide ion at m/z 826.46259 for MH3^3+^ corresponding to the mass of the peptide KGVNLPGAAVDLPAVSEKDIQDLK from PKM2 protein; (**D**) Alignment of the 11 peptides identified on the sequence of the PKM2 protein (identifier: P14618-1); (**E**) HEK293 cells transfected with human PKM2-flag/pCMV-2B (lane 2, 4) or with pCMV-2B plasmid (lane 1, 3). Cell lysates were blotted with the mouse anti-flag IgG Ab (M185-3L, MBL) (lane 1, 2; 1:2000 diluted, final conc. = 0.5 ug/mL) or with H103 scFv Ab (lane 3, 4; 1:1 diluted, final conc. = 14.5 ug/mL), respectively. (**F**) Hypoxic HCCLM3 cell lysates were respectively immunoprecipitated by Ni- (lane 1, 2) or protein L (lane 3, 4) agroase without (lane 1, 3) or with the H103 scFv Ab (lane 2, 4). Immunoprecipitations were blotted with the commercial anti-PKM2 McAb (c-11) (1:500).

Both protein L and the Ni-NTA agarose-based scFv Ab immunoprecipitation products showed a dominant band with an apparent MW of 58 kDa (Figure 5A). The extracted protein that underwent LC-MS/MS analysis unambiguously identified 11 unique peptide sequences (Figure 5B, 5C, 5D), which matched the PKM2 protein (NCBI accession number: P14618-1), a cancer-preferentially-expressed M2 type isoform of pyruvate kinase [22–24]. For independent verification, we ectopically expressed the human PKM2/pCMV-2B plasmid (from Fudan University) in HEK293 cells and found that the H103 scFv Ab specifically bound to the exogenous PKM2 protein in Western blotting (Figure 5E). Direct blotting of H103 scFv immunoprecipitation using the commercial anti-PKM2 Ab (C-11) gave a specific band at 58 kDa (Figure 5F). These results indicated that the H103 scFv Ab specifically recognizes the PKM2 antigen, and the binding affinity of the H103 scFv Ab is fairly acceptable.

### Hypoxia induces PKM2 increased expression *in vitro*

**Figure 6:**
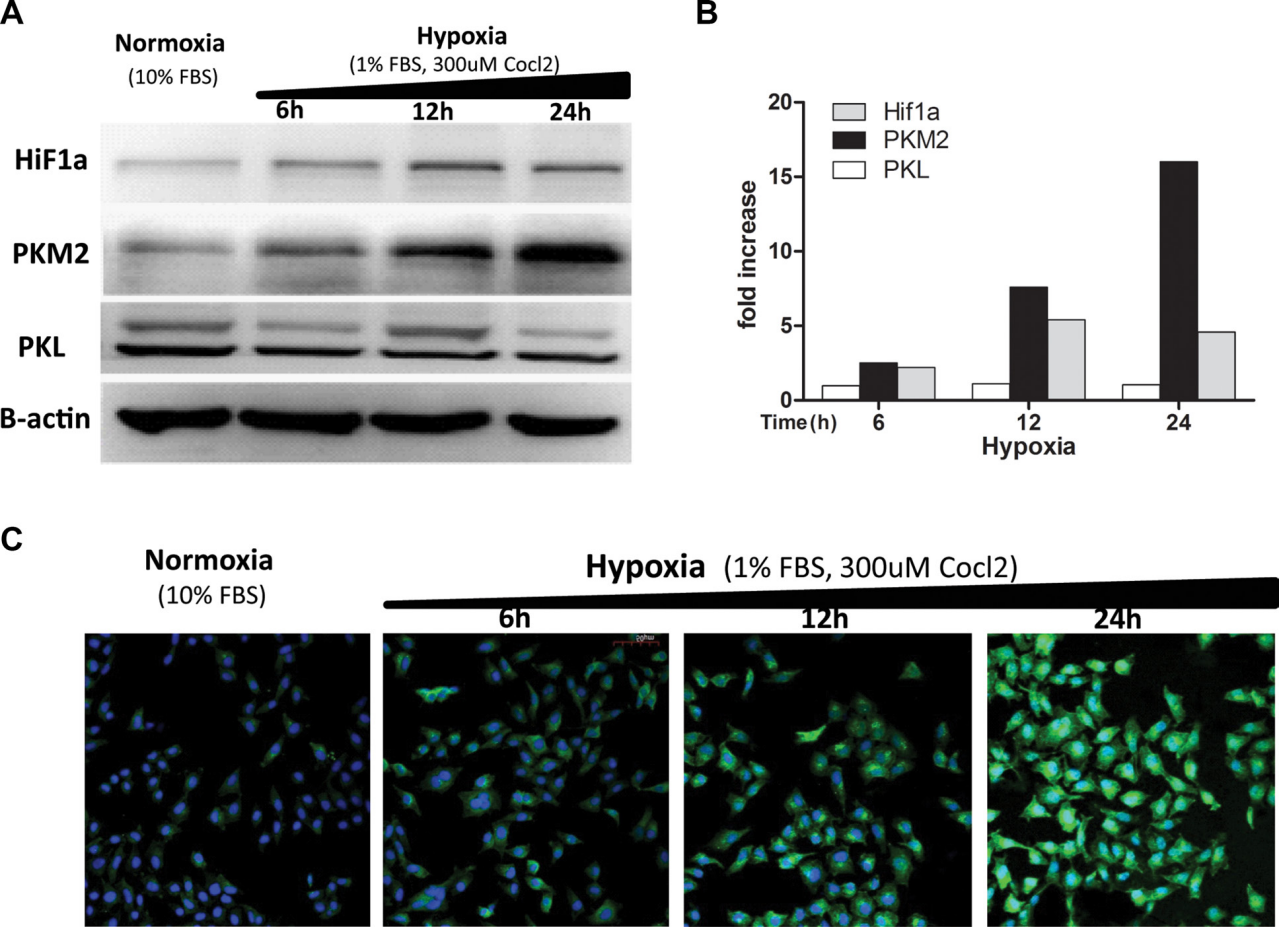
Hypoxia induces increased expression of the PKM2 protein (**A**) Different time hypoxic treated HCCLM3 cell lysates were blotted with the anti-PKM2(c-11) mAb (1:500), anti-PKL mAb (1:500), anti-Hif1a mAb (1:500), and anti B-actin mAb (1:500). Three independent experiments were performed and representative blot pictures are shown. (**B**) The graph presents the results from the quantitative analysis of expression levels of PKM2, PKL, and HiF1a determined via densitometry (reference to b-actin). The data collected include the following: means, *n* = 3. (**C**) HCCLM3 cells were fixed, permeabilized, and incubated with the H103 scFv Ab (1:1) overnight at 4°C followed by sequential staining with mouse anti-his (1:200) and AF488 conjugated anti-mouse (1:200). After rinsing with PBS/1M NaCl, the stain signals were visualized under confocal microscopy and representative images for three independent experiments are shown.

Although four isoforms of pyruvate kinase (PKM1, PKM2, PKL, and PKR) exist in mammal cells, only PKM2 and PKL were shown to exist in liver tissue [22–24]. As the PKM2 gene was shown to be transcriptionally activated by HIF-1a in different cell lines [22, 25], we checked their protein levels in liver cancer cells. Hypoxia-treated HCCLM3 cells presented a time-dependent increase (up to 16-fold) in the amounts of the PKM2 and HIF-1a protein, while the PKL protein level was not significantly elevated (Figure 6A, 6B). Similar effects were observed in 7721 and HepG2 cells (Figure S3B, S3C, S3D, S3E), but not in 7702 cells (data not shown). While hypoxia-induced PKM2 was markedly elevated, we did not find a clear-cut upregulation of HIF-1a. The level of the HIF-1a protein was rather reduced in HepG2 cells after 24 h hypoxic exposure (Figure S3C, S3E), which was in accordance with other reports [26]. Immunofluorescence analysis showed that the PKM2 staining signal was specifically elevated after 24 h hypoxic exposure while the PKL staining signal remain unchanged in HCCLM3, 7721, and HepG2 cells (Figure S3A). Again, a time-dependent increase in the staining of the PKM2 protein was markedly observed in hypoxia-treated HCCLM3 cells (Figure 6C). These results indicated that PKM2 is a hypoxia-stimulated protein highly expressed in liver cancer cells.

### Hypoxia-specific PKM2 antigen expression *in vivo*

**Figure 7:**
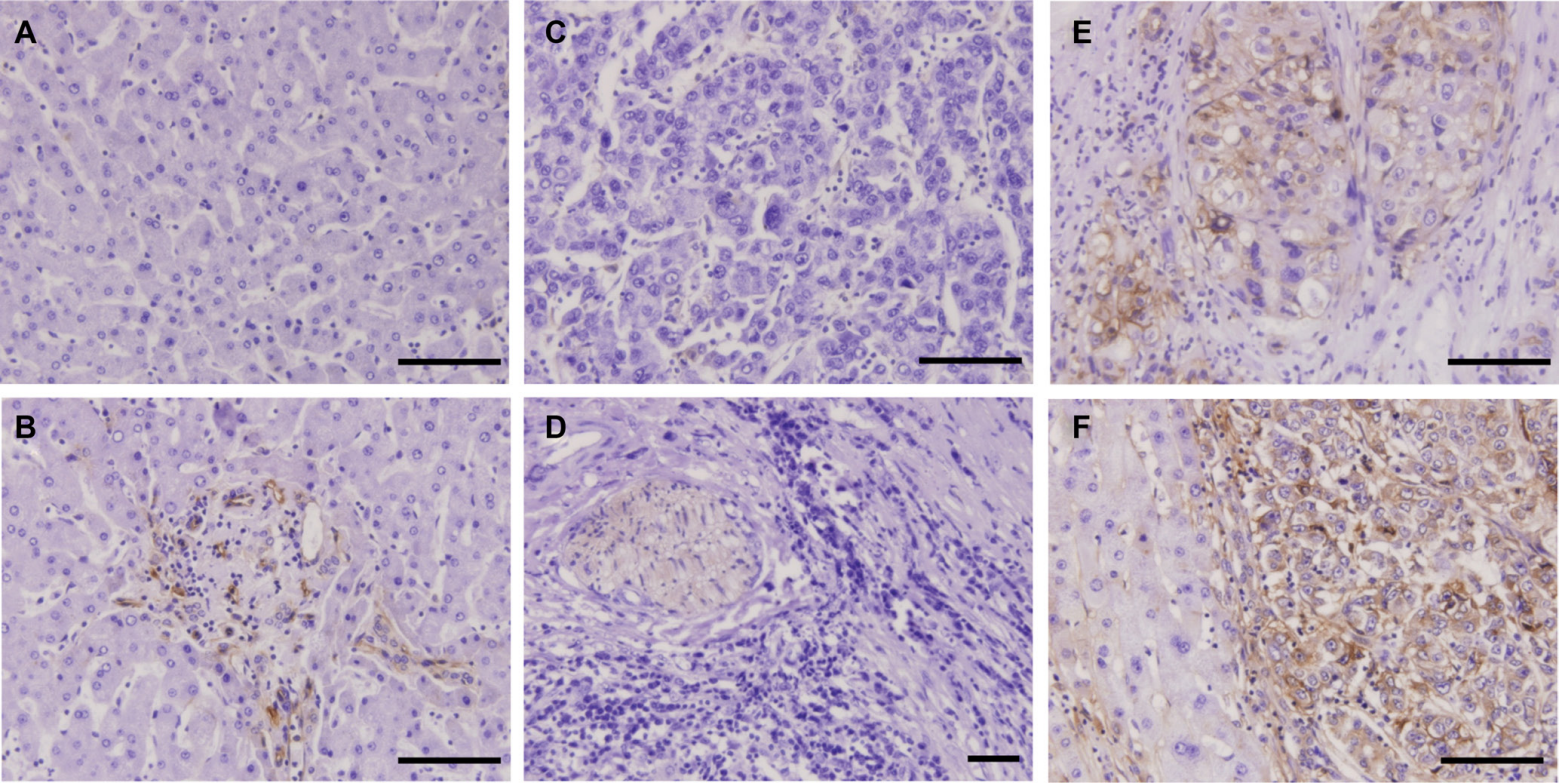
IHC staining of HCC tissues using the H103 scFv Ab Immunohistochemical staining of the H103 scFv Ab in MTA containing different grades of HCC tissues (94 cases), adjacent non-tumor liver tissues, and liver tissue sections (5 cases). Representative photomicrographs of PKM2 expression from two independent experiments are shown: (**A**) normal liver tissue stained with irrelevant scFv Ab E4B7S (0.1 mg/mL). H103 scFv Abs at matching concentration were respectively stained on normal liver tissue (**B**), poorly differentiated HCC tissue (**C**), well differentiated HCC tissue (**D**), and moderately differentiated HCC tissue is surrounded by pericancerous non-tumor liver tissue (**E**, **F**); Scale bars, 100 μm.

**Figure 8:**
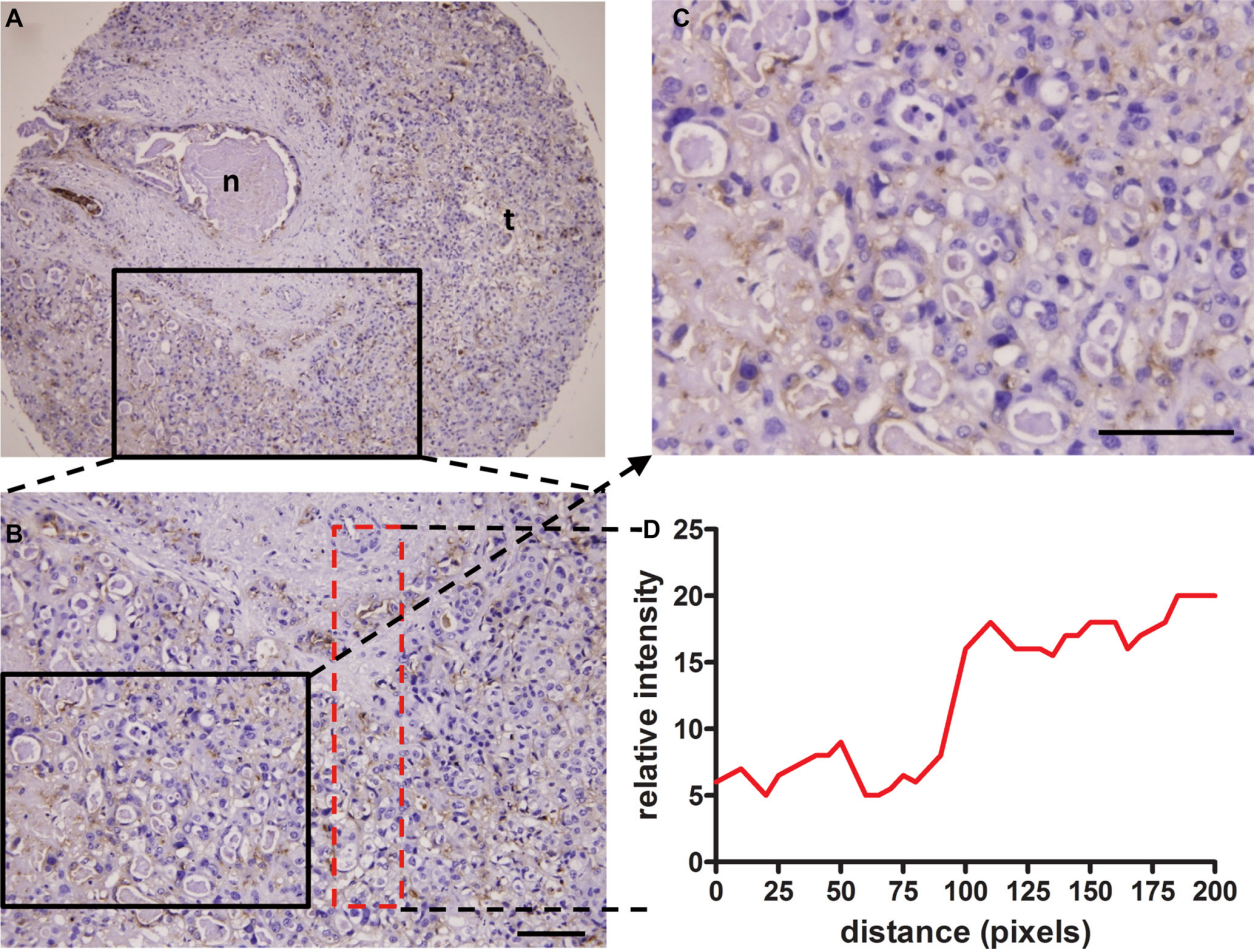
Perinecrotic staining pattern of the H103 scFv Ab in ICC tissue Immunohistochemical staining of the H103 scFv Ab in ICC MTA sections (100 cases) composed of a central bile canaliculus with a necrotic area inside (**A**), surrounded by ICC cells. Representative photomicrographs of PKM2 heterogenous perinecrotic expression from two independent experiments are shown (A, **B**). Staining intensity increased with the growth distance from the bile capillary (/blood capillary), as shown in detail on the magnified area (**C**) and graphically illustrated below as a distribution of staining signal relative to the distance from the bile capillary (**D**); n, necrosis; t, tumor cells. Scale bars, 100 μm.

IHC staining frequently revealed membranous/cytoplasmic staining of PKM2 with strong intensity in moderately and well differentiated HCC tissues, but entirely negative or weak staining of PKM2 in normal liver tissues and poorly differentiated HCC tissues (Figure 7). To determine the relationship between tissue hypoxia and PKM2 expression, HCC tissues with or without sinusoidal capillarization were paired-selected for staining. PKM2 was strongly stained in hypoxic regions, which indicated few blood vessels among different grades of HCC tissues. This sharply contrasted with paired HCC tissues containing numerous blood vessels, which only presented positive staining of PKM2 in vascular endothelial cells (Figure S4). Notably, hypoxic regions were mainly located in the center area of cancer cell nests, especially in moderately differentiated HCC tissues (Figure 7E, 7F, S4D). To further confirm this phenomenon, we selected a ICC tissue microarray with a necrotic area inside the central bile canaliculus for staining. Although PKM2 expression showed heterogenous and perinecrotic patterns in the whole ICC tissue, the positive staining intensity significantly increased with an increasing distance from the bile capillary (/blood capillary) (Figure 8). These results strongly indicated that hypoxia in hypovascular regions of liver cancer tissues induce the upregulation of PKM2 specific-expression.

### PKM2 can serve as a hypoxic marker in HCC and ICC

As PKM2 is highly expressed in hypovascular HCC and perinecrotic ICC tissues, we further evaluated the clinical relevance of PKM2 expression with patient characteristics data provided with MTAs. As shown in Table 2 and Table 3, tumor patients with moderately and well differentiated grades had significantly higher positivity for PKM2 than those with a poorly differentiated stage (88.41% vs 43.75% in HCC, *P* < 0.01; 74.47% vs 40% in ICC, *P* < 0.01). For ICC patients, tumors with an advanced stage (T3 + T4) had a significantly higher positivity for PKM2 than those with a lower stage (80% vs 28.89%, *P* < 0.01). However, in HCC patients, tumors with a lower stage (T1 + T2) had a significantly higher positivity for PKM2 than those with an advanced stage (84.72% vs 46.15%, *P* < 0.01). Tissue PKM2 positivity was not significantly different in ICC patients with regional lymph node or distant metastasis and those without metastasis (*P* = 0.1459, *P* = 0.1266 respectively). Strikingly, both HCC and ICC patients with no vascular invasion in their tumor tissues had a significantly higher positivity for PKM2 than those with vascular invasion (86.08% vs 26.66% in HCC, *P* < 0.01; 60.4% vs 22.22% in ICC, *P* < 0.05). Furthermore, a higher positivity of PKM2 expression was significantly detected in ICC patients with intra-tumoral necrosis than those without (91.66% vs 52.27%, *P* < 0.01). The perinecrotic high expression of PKM2 in ICC mirrors the situation in hypovascular HCC tissue, and agrees with the fact that increased expression of PKM2 in liver cancer cells is induced by hypoxia. All these results collectively support the potential of PKM2 serving as a hypoxia marker in HCC and ICC.

**Table 2:** Correlation of PKM2 expression with clinicopathological parameters from HCC patients

Parameter	PKM2		*P*
	Negatvie (*n* = 22)	Positive (*n* = 72)	
Sex			0.384
Male	18	64	
Female	4	8	
Ag			0.626
> 50	10	37	
≤ 50	12	35	
Differentiation (Grade,G)^*^			< 0.0001
Well	3	4	
Moderate	5	57	
Poor	9	7	
Unknown	5	4	
T Classification^**^			0.0017
T1 + T2	11	61	
T3 + T4	7	6	
Tx	4	5	
Regional lymph nodes status & Distant metastasis			---
N0 M0	17	68	
Nx Mx	5	4	
Intratumoral necrosis			0.3305
Yes	0	3	
No	22	69	
Vascular invasion			< 0.0001
Yes	11	4	
No	11	68	

* Differentiation: “well + moderate” vs “poor”;

** T classification: “T1 + T2” vs “T3 + T4”.

**Table 3:** Correlation of PKM2 expression with clinicopathological parameters in ICC patients

Parameter	PKM2		*P*
	Negatvie (*n* = 43)	Positive (*n* = 57)	
Sex			0.427
Male	23	35	
Female	20	22	
Ag			0.935
> 50	29	38	
≤ 50	14	19	
Differentiation (Grade, G) ^*^			0.0008
Well	5	9	
Moderate	7	26	
Poor	27	18	
Unknown	4	4	
T Classification^**^			< 0.0001
T1 + T2	32	13	
T3 + T4	11	44	
Regional lymph nodes status			0.1459
N1	10	21	
N0	33	36	
Distant metastasis			0.1266
M1	0	3	
M0	43	54	
Intratumoral necrosis			0.0097
Yes	1	11	
No	42	46	
Vascular invasion			0.0272
Yes	7	2	
No	36	55	

* Differentiation: “well + moderate” vs “poor”;

** T classification: “T1 + T2” vs “T3 + T4”.

## DISCUSSION

Conventional cell-based panning methods typically yield phage Abs specific for a wide range of undesired cell surface proteins, not just those that are associated with a defined cell phenotype as is the case with hypoxia in this study. In general, most hypoxia-responding proteins are not membrane antigens in cancer cells such as markers of angiogenesis, stromal antigens, and intracellular proteins released at sites of necrosis [21]. This brought difficulties to the original cell-based panning, especially when the targeted antigen is lowly expressed. Previous reports utilized cell sorting methods to isolate Abs bound with the desired molecular phenotype [15], [20]. Although successful, these approaches are limited by the prerequisite of known markers or Cell Tracker dye available to label the defined phenotype, besides the complexity of the sorting technique and expensive equipment. Therefore, selecting appropriate cell lines that express all the normal ‘housekeeping’ proteins but lack the phenotype-associated antigen for use in library depletion steps is vital for hypoxia panning. For the selections in this study, aliquots of normoxic NIH3T3 and HCCLM3 cells were sequentially utilized to deplete the Ab library after positive selection of hypoxic HCCLM3 cells in the first step of panning (Figure 1). This allows the maximum depletion of scFv clones that are specific for other proteins naturally present on the cells, and also decreases the likelihood that desired scFv clones would be lost during the panning. More particularly, our method involves the optimization of washing conditions to achieve an acceptable signal/noise ratio (Figure S2) and decent positive yield (Figure 2A). All of these efforts finally produced the isolation of hypoxia-binding scFv Abs.

Low affinity of isolated clones are a common issue with cell-based selections that can sometimes be resolved by combining less stringency in the early rounds of selection with greater stringency in the later rounds. We explored this attempt but failed to isolate hypoxia specific scFvs with high affinity (data not shown), suggesting that this may be related to the scFv Ab itself contained in prime library. Actually, the affinity of the H103 scFv Ab bound with hypoxic HCCLM3 cells is quite acceptable (2.08 × 10^8^ M^−1^), and it also works well in Western blot (Figure 5E), IHC staining (Figures 7, S4, 8), and scFv-based immunoprecipitation (Figure 5A). Similar to previous findings [12], immunoprecipitation using Ni-NTA agarose, which captures H103 scFv via its (His)6 tag, led to unspecific precipitation of many intracellular proteins, that complicated the antigen identification by LC-MS/MS analysis. In contrast, immunoprecipitation with protein L led to a much cleaner but relatively weak immunoprecipitates (Figure 5A). Early study reported that protein L is only effective in binding with certain subtypes of kappa light chains [27]. As the VL sequence of H103 scFv matches the IGλV6-57 germline gene, the successful immunoprecipitation by protein L in this study, suggested that specific binding of protein L with the tertiary structure of the H103 scFv Ab may exist, which accounts for their selective binding.

Pyruvate kinase, as a cytoplasmic enzyme, is generally not expected to localize at the cell surface. However, the specific binding of the H103 scFv Ab to hypoxic cells (Figures 2C, 2D, 3B, 4A, 4C; Table S2) and its antigen PKM2 identification based on cell surface-biotinylation (Figure 5) in this study, both support that a fraction of PKM2 protein did present on the hypoxic liver cancer cell surface. This result is in line with a previous report that a cryptic signal peptide directs the transport of pyruvate kinase to the cell surface [28]. In addition, PKM2 was shown to bind with tumor endothelial marker 8, which is located on the endothelial cell surface [19]. It also binds with the cell surface adhesion molecule CD44 in hypoxic cancer stem cells [29]. Furthermore, accumulating studies show that apoptotic agents or hypoxic stress can induce PKM2 translocation to the nucleus, suggesting its dynamic subcellular trafficking in response to different microenvironmental stimuli [30–32]. These observations together with our present results collectively suggest that hypoxia may induce PKM2 translocation to the surface of cancer cells and binding with the H103 scFv Ab triggers endocytosis. This will benefit hypoxia-specific targeted therapies in liver cancer.

PKM2 is the prototypic isoform of pyruvate kinase, and is gradually replaced by the PKL isoform during liver cell differentiation [24]. Previous studies reported isoform shifts from L to M2 in hepatocarcinogenesis [33, 34], and from M1 to M2 in other tumor formations [25, 35]. We found in this study that hypoxia induces increased expression of the PKM2 protein, but not PKL or PKM1 in liver cancer cell lines (Figures 6, S3). This mirrors the early observation that PKM2 mRNA is up-regulated in hypoxia-treated hepatoma cells [36]. There are three possible mechanism underlying PKM2 overexpression in hypoxic cells. First, SP3 binding sites were found in the PKM promoter and expression of SP3 represses PKM promoter activity. Hypoxia removes the associated transcriptional repression by down-regulating SP3, and thereby activates PKM expression [37]. Second, a new hypoxia response element (HRE) was found in intron 1 of PKM2, but not PKM1. Hypoxia strongly mediates the transcription of PKM2 by enhancing the HIF1a DNA-binding activity and recruiting p300 to HREs [38, 39]. Third, PKM2, acting in a feedback loop, directly interacts with HIF1a and promotes transactivation of HIF1a downstream genes. The interaction between PKM2 and HIF1a is mediated by the PHD3-dependent hydroxylation of PKM2. Because both PKM and EGLN3 (encoding PHD3) are HIF1a target genes, the positive feedback loop that promotes HIF1a activity maintains overexpression of PKM2 and other glycolytic genes [39], which accelerates metabolic reprogramming of cancer cells and may be an alternative salvage pathway to supply ATP to promote hypoxic HCC growth or survival.

The reason why PKM2 is highly expressed in moderately differentiated HCC, but weakly expressed in poorly differentiated HCC (Figure 7E, 7F; Table 2) is largely unknown. Many studies revealed that hypoxia can inhibit tumor cell differentiation and may maintain undifferentiated “stem cell-like” phenotypes, mediated by HIF-controlled stem cell genes up-regulation and differentiation genes down-regulation [40, 41]. Thus, hypoxia-induced PKM2 high expression should be markedly observed in poorly differentiated HCC. However, in this study, we only found that PKM2 is highly expressed in moderately differentiated HCC (Figure 7E, 7F; Table 2). This is somewhat in line with two previous findings: one is that PKM2 expression is upregulated in PMA-induced megakaryocytic differentiation and that PKM2 silencing inhibits its differentiation in leukemia [36]. The other is the interaction of PKM2 with Oct4, a major regulator of self-renewal and differentiation in stem cells, decreases the transcriptional activity of Oct4 for ‘stemness’ maintaining of glioma stem cells and thus induces differentiation [37]. Nevertheless, some contradictory results need attention, with one study demonstrating the high PKM2 expression in poorly differentiated esophageal squamous cell cancer tissues [38], another showing that PKM2 promotes OCT4 expression in hypoxic hESCs and also enhances its transcriptional activity by binding to OCT4 [42]. Given that PKM2 can promote cell proliferation by interaction with a lot of molecules (e.g. histone 1, STAT3, b-catenin) and direct cell differentiation via its interaction with Oct4 [22, 43], it is tempting to assume that PKM2 might function as a key balancer between a hypoxia-promoted growth/survival phenotype and a hypoxia-mediated dedifferentiation phenotype in HCC, both of which need PKM2 high expression to activate numerous transcription factors downstream of HiF1a. This postulation is currently being investigated in our laboratory.

The potential role of PKM2 as a hypoxia marker for HCC and ICC is indicated by its significant associations with necrosis (Figure 8; Tables 2, 3). Although showing heterogeneous distribution, high expression of PKM2 in ICC tumors is virtually restricted to regions directly adjacent to areas of necrosis. Furthermore, we found that PKM2 high expression in HCC is closely associated with aggressive pathological features, including high tumor grade and lymph node or distant metastasis. These findings are in accord with recently reports that overexpression of PKM2 contributes to the aggressiveness and poor prognosis of HCC [44, 45]. Taken together, it may be plausible that hypoxia and tumor necrosis are biologically associated, and PKM2 prognostically serves as a hypoxia marker for invasive liver cancer. To the best of our knowledge, this is the first report describing that PKM2 is a hypoxia marker in cancers. Further work with large cohorts of cancer patients is needed to confirm this finding. The present study also showed that PKM2 expression is correlated with the absence of vascular invasion in HCC and ICC (Figure S4; Tables 2, 3). This finding appears to be in contradiction with recent reports showing the associations of PKM2 overexpression with microvascular invasion in HCC tumors [34, 44, 45] and with a higher number of MVD in human biliary tract cancer [46]. The discrepancy between our results and others may be because of the limited cases of tissue array included in this study. Further study with expanded liver cancer patients and function investigation of PKM2 on liver cancer angiogenesis are needed.

In summary, this study, for the first time, developed a human scFv Ab H103 bound specifically with hypoxic liver cancers cells, and found that its antigen PKM2 is a promising biomarker for hypoxia in HCC and ICC tissues. Our findings indicated that direct selection of the phage Ab against the tumor hypoxia phenotype can provide an efficient approach for the discovery of a tumor metabolic marker at the protein level.

## MATERIALS AND METHODS

### Cell culture and hypoxia treatment

Human liver cancer cells 7721, HepG2, HCCLM3, hepatocyte cell 7702, embryonic kidney cell 293, and mouse embryo fibroblast 3T3 were obtained from China Infrastructure of Cell Line Resources (http://cellresource.cn/). Cells were grown in Dulbecco's Modified Eagle's Medium (DMEM) supplemented with 10% (v/v) fetal bovine serum (FBS) at 37°C with 5% CO_2_. For hypoxia treatment, cells were exposed to 100–300 μM CoCl_2_ in DMEM containing 1% FBS for 6–24 hours depending on the specific assay aim.

### Construction and identification of scFv Ab library

The human scFv Ab library was constructed as previously described [16]. Briefly, human Ig VH(G + M), VLκ, and VLλ gene family pools were PCR amplified from PBMC of liver cancer patients. Gel-purified gene mixtures of IgVH(G:M = 1:1), IgVL(κ:λ = 2:1), and (Gly4Ser)_3_ (H-κ, H-λ) linker were three-fragment assembled into a scFv repertoire using splicing overlap extension PCR, re-amplified to append restriction sites (Sfi 1 & Not 1), then subcloned into the pCANTAB 6 his plasmid (substitute E-tag in pCANTAB 5E to 6 × His tag), and electro-transformed into E. coli TG1 to generate a scFv library in bacterial form.

Gene diversity in the library was determined by sequencing 30 randomly selected clones and was analyzed using the IMGT/V-QUEST online program (http://imgt.cines.fr/textes/vquest/). Phage Abs were prepared using M13K07 helper rescue followed by PEG-NaCI precipitation. The size and titer of the scFv Ab library were measured as previously described [17, 18].

### Panning and screening of hypoxia binding scFv Abs

(1) Subtractive and positive selections: The procedure (Figure 1) was similar to that previously described [10]. Positive selection was performed on hypoxia-treated (300 uM Cocl_2_) HCCLM3 cells by incubating 1 × 10^7^ PBS/EDTA-detached cells with 3 × 10^8^ pfu of the library at 4°C for 1 h. Bound phages were stripped by glycine/NaCl buffer (pH 2.5) then neutralized with 1 M Tris (pH 7.4). Subtractive selection was performed by incubating 4 × 10^8^ 3T3 and HCCLM3 cells with 4 × 10^7^ pfu of the phage library. The final sub-library in each round was rescued again and subjected to the next round of panning. (2) Flow cytometry-based clone screening: phage-scFv and the soluble scFv Ab (periplasmic extract of scFv-phage-infected HB2151 E. coli cells) from individual clones were rocking-incubated with hypoxic cells (PBS/EDTA detached) at 4°C for 1 h. Bound phage scFv Abs were detected by adding a mixture of biotinylated anti-M13 McAb (1:600, Sigma) and streptavidin-AF488 (1:1000, Molecular Probes). Bound soluble scFv Abs were detected by addition of a mixture of mouse anti-his mAb (1/300) with AF488 conjugated goat anti-mouse pcAb (1/1000, Molecular Probes). The fluorescence was measured in a FACScalibur (BD Biosciences). E4B7S (anti-γ-seminoprotein) and H18S (anti-CD147) scFv Abs [17, 18] were used as controls.

### Expression and purification of the scFv Ab

The scFv gene was cloned into the pET28a-His plasmid (Novagen) by Nco I+ Not I site. Transfected BL-21 (DE3) pLysS E. coli cells were shake cultured in LB medium (1 mM IPTG, 100 ug/ml ampicillin, 34 ug/ml chloramphenicol) for 6 h at 37°C. Bacteria pellets were harvested and sonicated in lysis buffer (50 nM Tris-HCl, 0.1 mg/ml lysozyme, 1 mM PMSF, pH 8.0). Inclusion bodies were completely solubilized in 8 M urea denaturation buffer, centrifuged at 30,000 × g for 20 min, and the supernatant was loaded on a His Bind resin column (GE Healthcare). The bound scFv Ab was eluted and then dialyzed against refolding buffer I (50 mM Tris-HCl, 2 M urea, 2 mM reduced glutathione, 0.2 mM oxidized glutathione, pH 8.0) and refolding buffer II (50 mM Tris-HCl, 5 mM EDTA, pH 8.0). The scFv protein was concentrated using an Ultrafree-4 centrifugal filter unit (Millipore) and its purity was analyzed by SDS-PAGE followed by Coomassie blue staining.

### Affinity purification of the cognate antigen

Hypoxic HCCLM3 cells were surface-labeled with 0.1 mg/ml Sulfo-NHS-LC-biotin (Pierce). After washing with PBS-glycine (50 mM), cells were PBS/EDTA detached and the lysates were depleted by incubation with either protein L (Santa Cruz) or Ni-NTA-agarose (Quiagen) for 1 h. Depleted lysates were incubated with the scFv Ab (4 ug per ml) at 4°C overnight and the immune complexes were captured by protein L or Ni-NTA agarose. The captured immune complexes were washed with lysis buffer or eluted using 200 mM imidazole, and then resolved by 1D SDS-PAGE in duplicate. One gel was blotted with HRP conjugated streptavidin (Jackson), the other gel was stained with Coomassie blue, and the specific protein bands were excised with reference to Western blot.

### Sample preparation and LC-MS/MS analysis

The excised gel bands of interest were reduced with 25 mM of DTT and alkylated with 55 mM iodoacetamide. In gel digestion was then carried out with sequencing grade modified trypsin in 50 mM ammonium bicarbonate at 37°C overnight. The peptides were extracted twice with 0.1% trifluoroacetic acid in 50% acetonitrile aqueous solution for 30 min. Tryptic peptides were re-dissolved in 50 μL 200 mM Tetraethylammonium Bromide, and 2 μL TMT sixplex labeling reagent (Thermo) was added to each sample. The reaction was incubated for 1 hour at RT. Then, 0.5 μl of 5% hydroxylamine (pH 9) was added to the reaction mixture and incubated for 15 minutes to quench the reaction. Equal amounts of labeled samples from both immunoprecipitations were combined and analyzed by LC-MS/MS.

For LC-MS/MS analysis, the TMT-labeled peptides were separated by a 65 min gradient elution at a flow rate 0.250 μl/min with a Thermo-Dionex Ultimate 3000 HPLC system, which was directly interfaced with a Thermo Scientific Q Exactive mass spectrometer. The analytical column was a home-made fused silica capillary column (75 μm ID, 150 mm length; Upchurch) packed with C-18 resin (300 Å, 5 μm, Varian). Mobile phase A consisted of 0.1% formic acid, and mobile phase B consisted of 100% acetonitrile and 0.1% formic acid. The Q Exactive mass spectrometer was operated in the data-dependent acquisition mode using Xcalibur 2.1.2 software. There was a single full-scan mass spectrum in the orbitrap (400–1800 m/z, 60,000 resolution) followed by 10 data-dependent MS/MS scans at 27% normalized collision energy (HCD). The MS/MS spectra were searched against the human.fasta from UniProt (March 19, 2014) using an in-house Proteome Discoverer (Version PD1.4).

### Evaluation of internalization by confocal microscope and flow cytometry

Cells were incubated with either phage-scFv or soluble scFv Abs in serum-free DMEM at 37°C for 1 h. After washing with cold PBS/1M NaCl to remove surface-bound scFvs, cells were fixed in 3.7% (w/v) paraformaldehyde at RT for 10 min. To check the uptake of phage-scFvs, cells were blocked in 2% (v/v) goat serum, permeabilized with 0.2% (w/v) saponin for 15 min, incubated with mouse anti-M13 Ab (1:400) at RT for 1 h, washed three times with 1% BSA/PBS, and finally detected after 1 h incubation with AF488-conjugated anti-mouse Ab (1:1000). The uptake of soluble scFv Abs was similarly detected but with mouse anti-His (1:300) as the prime Ab. After nuclear staining with DAPI (1:1000), the cells were mounted and images were acquired using an Olympus FV 1000 laser scanning confocal microscope. The binding of scFv Abs at 4°C was checked as described above, without PBS/1M NaCl rinsing and saponin-permeabilization.

For flow cytometry-based uptake measurement, the soluble scFv Ab (1:20), mouse anti-His (1:300), and goat anti-mouse-AF488 Ab (1:500) were pre-complexed in serum-free DMEM at 20°C for 30 min (scFv-AF488 complex) and then incubated with cells at 37°C for 10 min-1 h. The uptake of phage-scFvs was measured by addition of a pre-complex consisting of phage-scFv Abs (1:30), biotinylated anti-M13 McAb (1:600), and streptavidin-AF488 (1:1000). Cells were then trypsinized, washed, and immediately live-analyzed. Controls without scFv Abs were included in all experiments. The binding of the pre-complexes with PBS/EDTA- or Trypsin/EDTA detached cells at 4°C were similarly measured as described above.

### Western blotting

Hypoxic treated or plasmid-transfected cells were washed with cold PBS and lysed in standard lysis buffer. The protein concentration was measured using the BCA protein assay. Equal amounts of lysate proteins were resolved by SDS-PAGE, blotted onto PVDF membranes, and then analyzed with the different Abs indicated in the text.

### IHC staining on tissue microarrays

Tissue microarrays (TMAs) were obtained from US Biomax Inc. (http://alenabio.com). 5 cases of human normal hepatic tissues and 94 cases of HCC tissues (two sections per case) were included in TMA #LV2082. 100 cases of ICC tissues (1 section/case) were included in TMA #LV1004. Sections were deparaffinized in xylene, rehydrated in a graded alcohol series, and washed with PBS. Quenching with endogenous peroxidase was done by incubating the sections in 3% H_2_O_2_ in methanol for 10 min at RT followed by 3 PBS washes. Antigen retrieval was performed by placing the slides in 10 mM citrate buffer (pH 6.0) and microwaving them twice. Sections were blocked for 30 min at RT in 20% normal goat serum, then incubated with purified scFv Ab (1:10) overnight at 4°C. After PBST washing, sections were incubated with biotin-conjugated anti-his McAb (1:50, Qiagen) for 30 min at RT. After 3 PBST washes, a mixture of avidin DH and biotinylated horseradish peroxidase H was applied for 30 min by using the Vectastain Elite ABC kit (Vector Laboratories). Detection of bound HRP was performed with ImmPACT™ DAB for 5 min. Control sections were treated as described above except for the incubation with scFv Ab.

### Statistical analysis

All data derived from at least three experiments were analyzed using GraphPad Prism 5 software. The student *t*-test was performed to compare two groups, and *p* ≤ 0.05 was considered significant. The chi-squared (χ^2^) test was used to evaluate the association of antigen expression with clinicopathological parameters.

## SUPPLEMENTARY MATERIALS FIGURES AND TABLES



## Abbreviations

HCC: hepatocellular carcinoma
ICC: intrahepatic cholangiocarcinoma
PKM2: M2 splice isoform of pyruvate kinase
scFv: single-chain Fv
Ab: antibody
Ig: immunoglobulin
IHC: immunohistochemical
HIF-1α: hypoxia inducible factor 1α
CA-IX: carbonic anhydrase IX
LC–MS/MS: liquid chromatography tandem mass spectrometry
