# Supplementary material for: Phage display library selection of a hypoxia-binding scFv antibody for liver cancer metabolic marker discovery
Source: Oncotarget. 2016 May 18;7(25):38105–21. doi: 10.18632/oncotarget.9460 (PMC5122375; doi:10.18632/oncotarget.9460)
Supplement: Supplementary file 1 [file oncotarget-07-38105-s001.pdf]

Phage display library selection of a hypoxia-binding scFv antibody for liver cancer metabolic marker discovery

Supplementary Materials

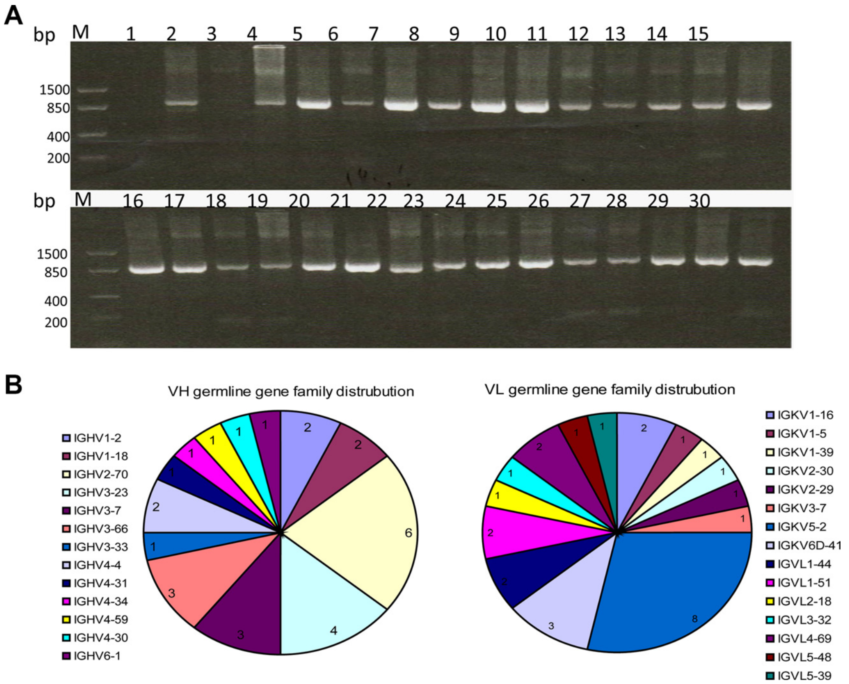

Supplementary Figure S1: Identification of the human scFv Ab library. (**A**) Evaluation of scFv gene-recombinant percentage. Gel electrophoresis analysis of PCR-amplified products of random-selected clones using 5′ and 3′ sequencing primers to the pCANTAB 6xhis plasmid. (**B**) IMGT V-QUEST analysis of the germline gene family distribution (IgVH, left; IgVL, right) matches the variable domain sequences of random-selected clones (identity > 90%).

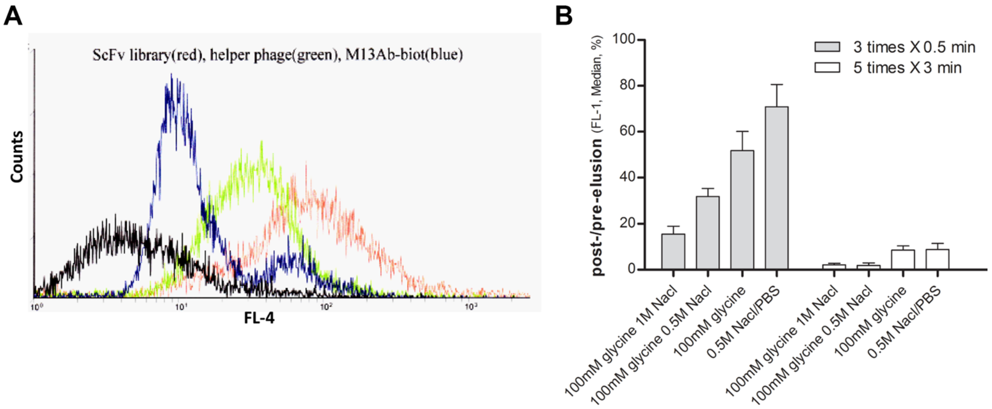

Supplementary Figure S2: Optimization of the stripping parameters for library selection. (**A**) PBS-EDTA detached HCCLM cells were shake incubated at 4°C for 1 h with the primer phage scFv library or helper phage M13K07 at the same titer. The binding was measured by flow cytometric analysis (biotinylated anti-M13 McAb, 1:600, streptavidin-AF647, 1:1000). (**B**) The bound phage library was washed for different times with different stripping buffers: (1) 100 mM glycine/1 M NaCI, pH 2.5, (2) 100 mM glycine/0.5 M NaCI, pH 2.5, (3) 100 mM glycine, pH 2.5, (4) 0.5 M NaCI/PBS, pH 7.0. Residual binders on the cell surface were compared by flow cytometric measurement.

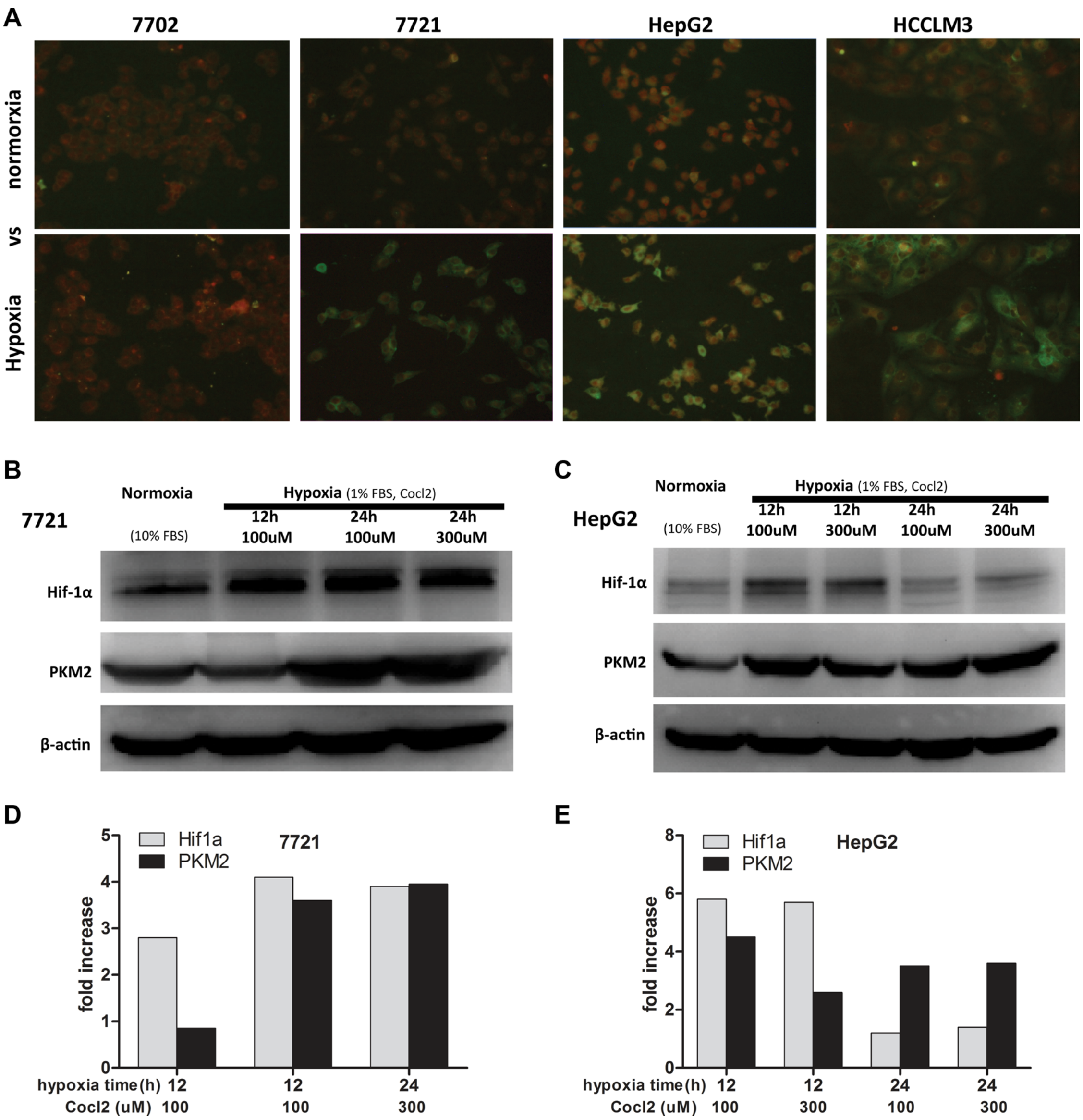

Supplementary Figure S3: Effect of hypoxia on expression levels of the PKM2 protein. (**A**) Hypoxic treated liver cancer cells were fixed, permeabilized, and stained for PKM2 with the mouse anti-PKM2(c-11) mAb (1:500) along with the AF488 conjugated secondary Ab (1:2000). Slides were then stained with the rabbit anti-PKL Ab (1:500) and then with the AF647 conjugated anti-rabbit Ab (1:2000) for 45 minutes. Double staining signals were visualized under a fluorescence microscope and representative images for three independent experiments are shown. Negative controls were performed by omitting the corresponding primary Ab and adding secondary IgG isotype Ab (× 400). (**B**, **C**) Total lysate of 7721 and hepG2 cells were respectively blotted with the anti-PKM2(c-11) mAb (1:500), anti-Hif1a mAb (1:500), and anti B-actin mAb (1:500). Three independent experiments were performed and representative blot pictures are shown. (**D**, **E**) Expression levels of PKM2 and HiF1a (reference to b-actin) were determined via densitometry (reference to b-actin). The data collected include the following: means, n = 3.

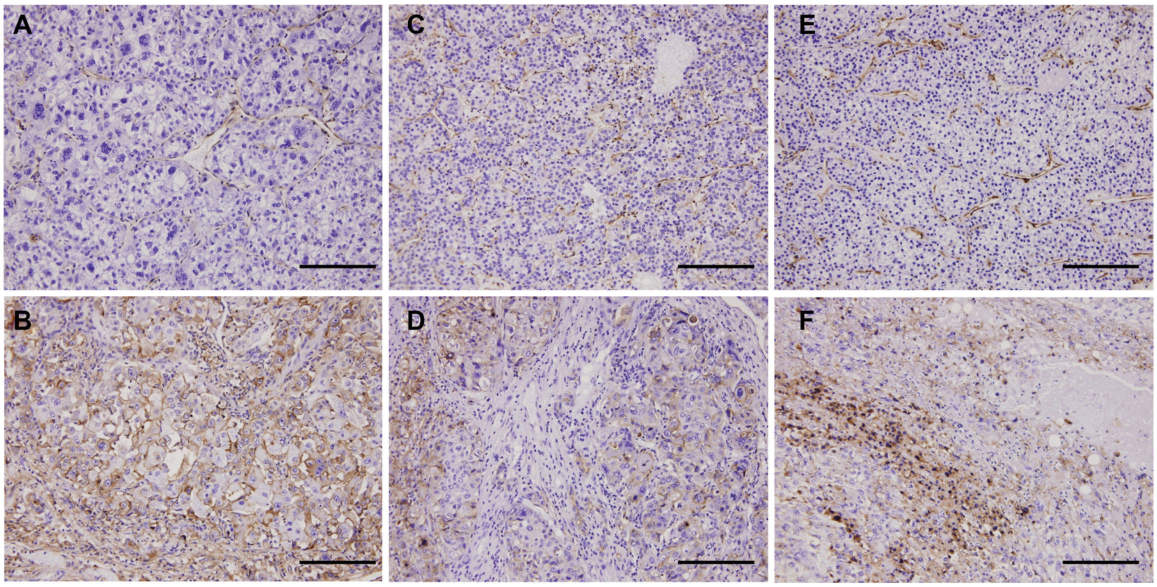

Supplementary Figure S4: Hypovascular staining pattern of the H103 scFv Ab in HCC tissue. HCC tissues with (**A**, **C**, **E**) or without (**B**, **D**, **F**) sinusoidal capillarization were paired-selected to stain with the H103 scFv Ab. Representative photomicrographs of PKM2 expression from two independent experiments are shown for poorly differentiated (A, B), moderately differentiated (C, D) and well differentiated (E, F) HCC tissues. Scale bars, 100 μm.

Supplementary Table S1: Germline gene analysis of seven unique hypoxia-binding clones

Clone	VH	JH	VD	$(Gly_4Ser)_3$ linker	VL	JL
H103	IGHV4-4*02	IGHJ4*02	IGHD3-22*01		IGLV6-57*02	IGLJ2*01
H114	IGHV4-4*02	IGHJ4*02	IGHD3-22*01		IGLV6-57*02	IGLJ2*01
H119	IGHV4-59*01	IGHJ5*02	IGHD6-19*01		IGKV3-15*01	IGKJ2*01
H117	IGHV3-23*01	IGHJ5*01	IGHD2-2*02		IGLV6-57*02	IGLJ2*01
H108	IGHV3-30*13	IIGHJ2*01	IGHD2-8*01		IGLV6-57*02	IGLJ3*01
H115	IGHV3-23*01	IGHJ3*02	IGHD2-21*01		IGKV3-20*01	IGKJ2*01
H121	IGHV3-13*01	IGHJ4*02	IGHD2-2*02		IGKV3-11*01	IGKJ4*01

Supplementary Table S2: Comparison of the hypoxia-binding of H103 scFv Ab with hypoxia-marker Ab GT12

Cell line	Origin	E4B7S		H18S		H103		GT12	
		Normoxia	Hypoxia	Normoxia	Hypoxia	Normoxia	Hypoxia	Normoxia	Hypoxia
7721	Human hepatoma cell	30.34	38.75	161.00	552.41	140.90	723.55	256.02	2372.23
HepG2	Low metastatic human HCC cell	29.25	34.90	101.86	331.45	67.15	546.62	289.22	2603.05
HCCLM3 (LM3)	HCC cell with high potential lung metastases	20.03	31.17	164.16	389.27	167.11	1172.39	268.94	3130.16
HL-7702 (L02)	Human immortalized hepatocyte cell	12.00	26.44	140.12	283.59	80.38	96.12	241.19	496.63
NIH/3T3	Mouse embryo fibroblast	13.71	17.25	18.24	19.09	11.26	17.55	32.47	59.48

*The bindings of H103, H18S (negative control) and E4B7S (unrelated control) scFv Abs were determined with the mixture of mouse anti-his (1/300) Ab and AF647 conjugated goat anti-mouse IgG (1/300). The bindings of hypoxia-marker Ab GT12 (mouse full-length IgG targeting Carbonic Anhydrase IX) were only detected with AF647 conjugated goat anti-mouse IgG (1/300). Mean values of median fluorescence intensity from three independent experiments are shown.
